# Tracing the stemness and malignant transition in a heritable colorectal cancer Lynch Syndrome by single-cell RNA-seq analysis

**DOI:** 10.3389/fimmu.2026.1722806

**Published:** 2026-05-25

**Authors:** Junfeng Xu, Jianlin Zhang, Yuhang Li, Zhiqin Wang, Qianru Li, Aijun Liu, Jianqiu Sheng, Ge Dong, Lang Yang, Zhigang Cai

**Affiliations:** 1 Senior Department of Gastroenterology, The First Medical Center of Chinese PLA General Hospital, Beijing, China; 2 State Key Laboratory of Experimental Hematology, Tianjin Medical University, Tianjin, China; 3 Department of Bioinformatics; The Province and Ministry Co-sponsored Collaborative Innovation Center for Medical Epigenetics, Tianjin Medical University, Tianjin, China; 4 Tianjin Key Laboratory of Inflammatory Biology; Department of Pharmacology, School of Basic Medical Science, Tianjin Medical University, Tianjin, China; 5 Department of Pathology, The Seventh Medical Center of PLA General Hospital, Beijing, China; 6 Department of Gastroenterology, The Seventh Medical Center of Chinese PLA General Hospital, Beijing, China; 7 Department of Rheumatology and Immunology, Tianjin Medical University Tianjin General Hospital, Tianjin, China; 8 Department of Hematology, Tianjin Medical University Tianjin General Hospital, Tianjin, China

**Keywords:** cell atlas, Lynch syndrome, mutation burden, single-cell RNA-sequencing, tumor immunity

## Abstract

**Background:**

Lynch Syndrome (LS) is an autosomal dominant disease characterized by germline heterozygous mutations in DNA mismatch repair (MMR) genes. High-risk LS patients may proceed to colorectal cancer (CRC). However, the drivers or biomarkers of LS benign colon tissue approaching malignant CRC are not completely understood. This study aimed to understand the molecular and cellular changes during malignant transition in LS.

**Methods:**

Single-cell RNA sequencing (scRNA-seq) was used to analyze paired fresh biopsy samples from 3 LS patients (carcinoma vs. para-carcinoma, labeled as LS-CA vs. LS-paraCA). Single-nuclear RNA sequencing (snRNA-seq) was used to analyze a frozen biopsy sample of a LS patient. Datasets of Healthy controls and patients diagnosed with sporadic CRC (without LS-related germline or somatic mutations; labeled as nonLS-CRC) were downloaded from the open source. Integrative computational analysis was performed to conclude potential drivers of the malignant transition. Immuno-histo-fluorescence staining (IHF) were also performed for validating the proposed three key markers.

**Results:**

In the single-cell atlas, we observed an increase of primitive cancer stem-cells with high expression of biomarkers *CEACAM5, BACE2*, *GPRC5A* and *OLFM4* in the epithelium of the LS. Both infiltration of immune cells and pathways related to DNA repair biological activity in LS are dramatically increased in carcinoma compared to para-carcinoma. The mutation burden in LS is fundamentally elevated compared to that in healthy controls. Furthermore, T cell and macrophage-related tumor immunity in LS is readily mobilized in carcinomas compared to para-carcinoma.

**Conclusions:**

This study provides single-cell transcriptomic resource using affected tissues from patients with Lynch Syndrome and describes an integrative profile covering the alterations of cancer stem cell markers, mutation burden, and tumor immunity during the malignant transition from latency state to Lynch Syndrome and to colorectal cancer at the single-cell level.

## Highlights

**Figure 1:**
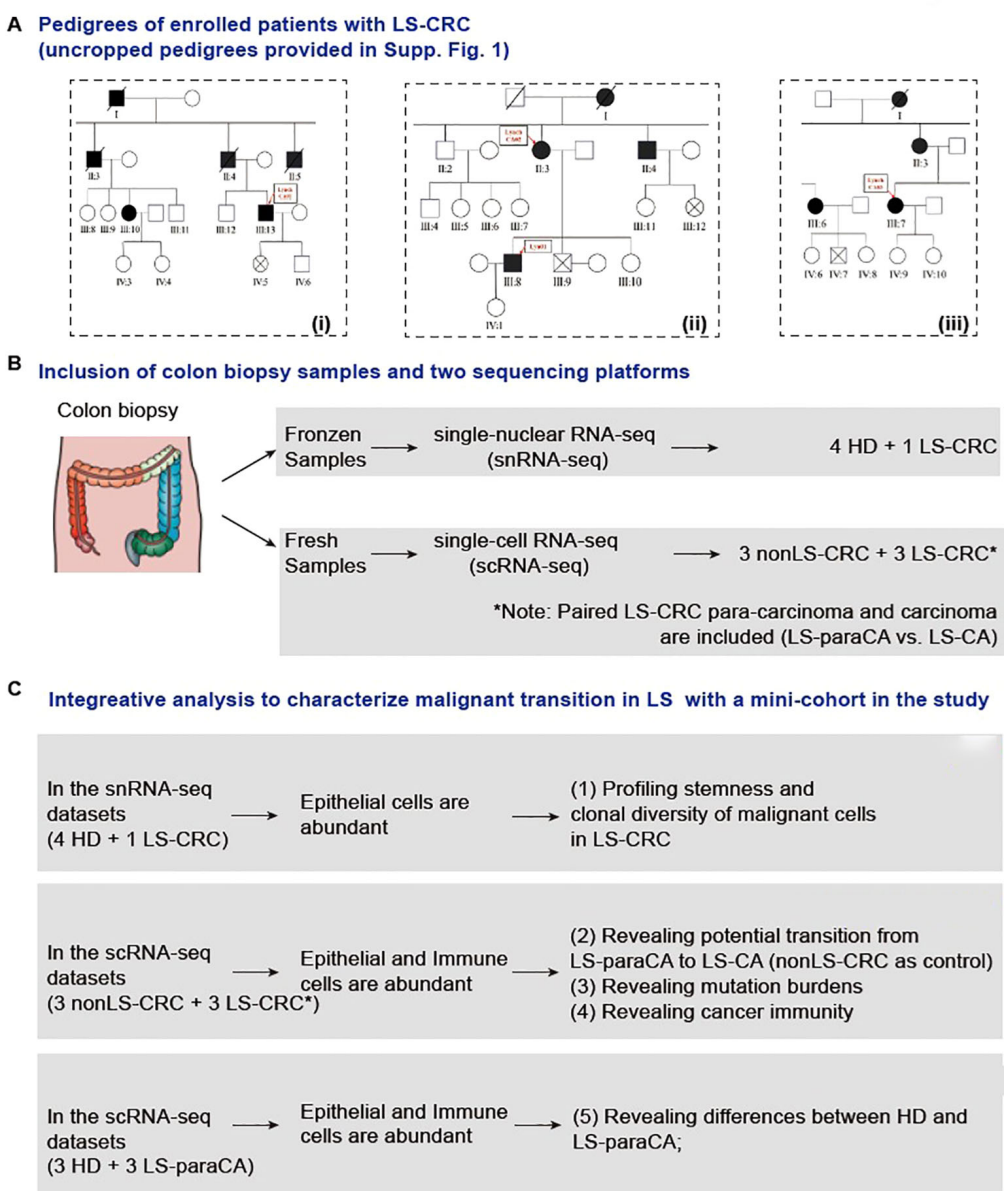
Enrollment of LS patients and overall design of the study. **(A)** Three family pedigrees of the LS patients who donated the samples for single-cell transcriptomic analysis. Cropped pedigree information is provided in the Main text. Germline mutations in *MLH1* were detected in Family (i) and (ii) while that in *MSH2* detected in Family (ii). See the **Supplementary Figure 1** for the complete version of the pedigree information. **(B)** Two different platforms were used in the study: snRNA-seq and scRNA-seq. **(C)** Various types of comparison were performed to characterize the malignant transition in LS.

**Figure 2:**
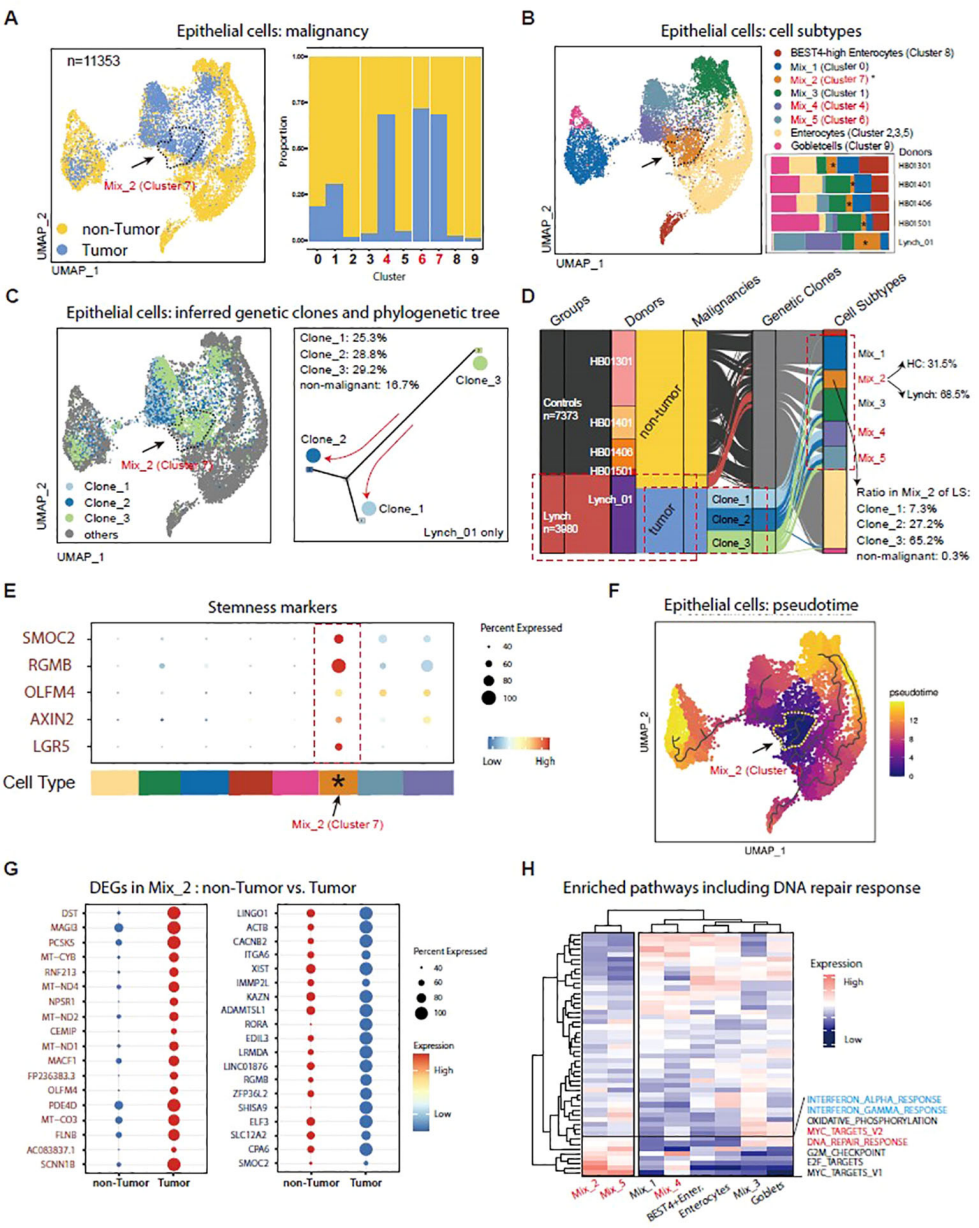
snRNA-seq analysis of a frozen colon biopsy sample with LS reveals a cluster of cancer stem cells. **(A)** Frozen colon biopsy samples were subjected for snRNA analysis (Healthy donors, N = 4; Lynch Syndrome, N = 1) and epithelial cells were filtered out for further study. Data were integrated by the Seurat and Harmony standard procedure. Clusters 0–9 were identified in a total of 11,353 cells (the Healthy Controls have 7,373 cells; the LS patient has 3,980 cells). The malignancy of the epithelial cells was inferred by the inferCNV and SCEVAN algorithm (two major compartments: tumor and no-tumor). The portion of tumor or non-tumor cells in each cluster was also illustrated (right panel). Note that the major contributor in Cluster 4, 6, and 7 is the tumor-like cells from the LS patient. **(B)** Each cluster of the epithelial cells was further annotated according to the combination of Seurat cluster identity and SCEVAN malignant identity. Mix_1 to 5 contain both tumor-like cells and non-tumor-like cells; Other clusters are marked as *BEST4^+^*-Enterocytes, Enterocytes and Goblet cells where much fewer tumor-like cells were assigned. Note that the epithelial cells from the LS patient are mainly classified into Mix_2, 4, and 5 tumor-like cells. **(C)** Three clones were inferred by SCEVAN algorithm. In the phylogenetic tree, Clone_1 and Clone_2 are closer and probably originated from Clone_3. **(D)** A Sankey plot shows overall lineage relationships in the epithelial snRNA-seq dataset. Note that the Mix_2 pool is dominated by cells from Lynch_01 and that in the Mix_2 pool Clone_3 is the most dominant clone. **(E)** The Mix_2 cells specifically express numerous colon epithelial stem cells signature markers such as *SMOC2*, *RGMB*, *OLFM4*, *AXIN2* and *LGR5*. Of note, all these intestine stem cell signature genes were reported as marker genes for cancer stem cells, especially *LGR5*. **(F)** Pseudotime trajectory analysis of the LS epithelium cells indicate the Mix_2 cells are the most primitive in development. **(G)** Top 36 DEGs (differentially expressed genes, both upregulated and downregulated) were highlighted when the non-tumor-like cells were compared with tumor-like cells in the Mix_2. **(H)** The Mix_2, 4, 5 epithelial cells all have enriched signaling pathways involved in DNA repair response and MYC targets (fonts in red) while Mix_4 and 5 manifest Interferon (IFN) alpha or gamma response (fonts in blue).

**Figure 3:**
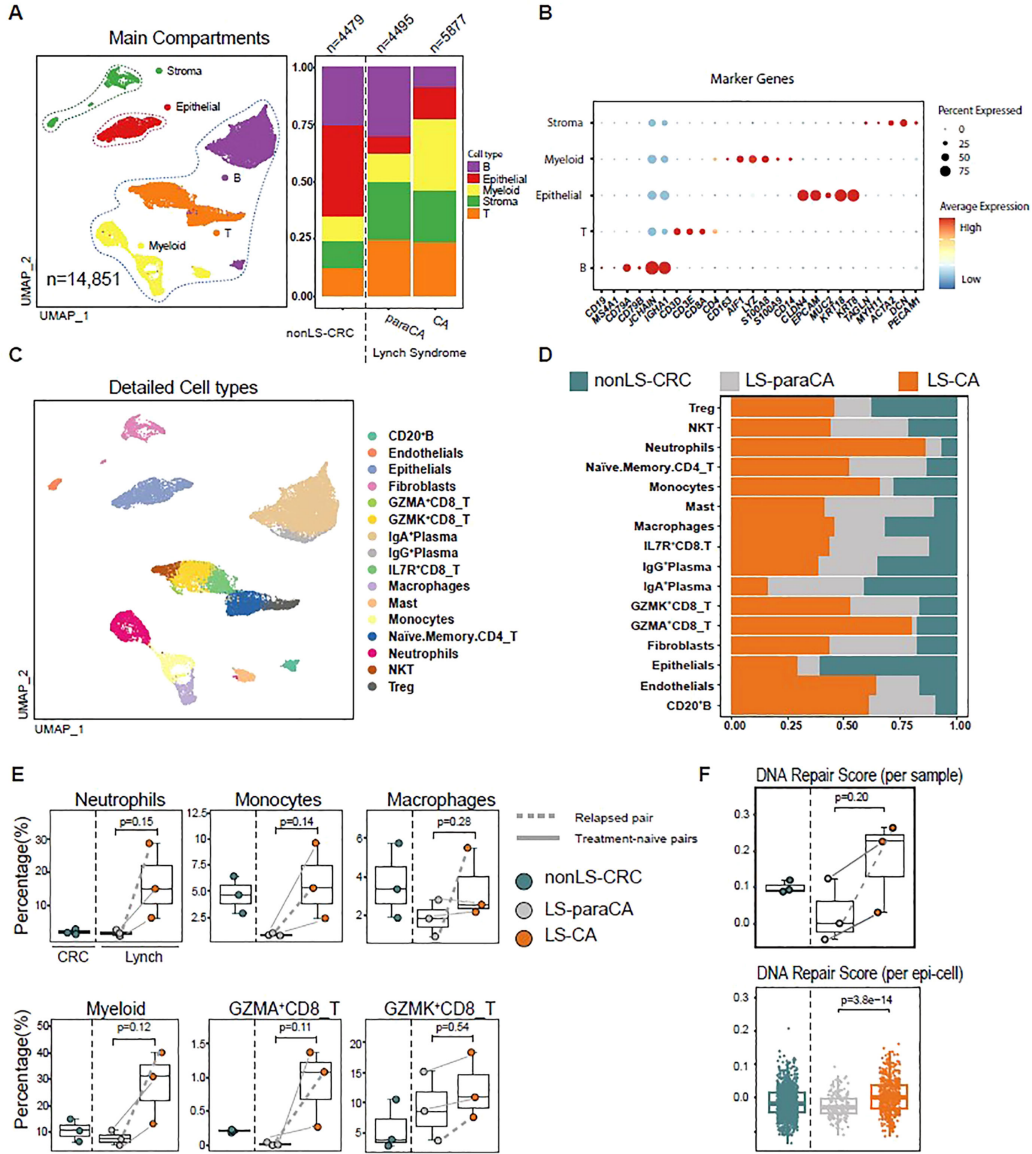
Distinct infiltration of immune cells in carcinoma and para-carcinoma tissues of LS **(A)** Fresh paired carcinoma (LS-CA) and adjacent para-carcinoma (LS-paraCA) biopsies of colon tissue were subjected to scRNA-seq analysis (N=3). Tumor samples from sporadic colorectal cancer without LS (nonLS-CRC; they are not diagnosed as LS or without LS-related somatic mutations) were downloaded from the public datasets and included as controls (nonLS-CRC, N=3). Cells were first grossly annotated as stromal, epithelial, or immune cells. Note that the LS-CA group showed increased infiltration of myeloid cells compared with LS-paraCA group, but T cell infiltration was comparable between the two groups. **(B)** Expression of classic markers for annotating the major 5 cell types. **(C)** Detailed annotations of 16 cell types in paired LS-CA and LS-paraCA samples. **(D)** Stacked bar plot showing the contribution of sample sources in each cell type. **(E)** Quantification of immune cell infiltration in LS-CA compared with LS-paraCA. Although no significant differences were observed, the fold-changes were approximately 2–10. Although the overall T cell infiltration was comparable, infiltration of GZMA^+^CD8*T* and *GZMK*^+^*CD8*T cells appeared to be higher in LS-CA than in LS-paraCA. Of the three LS patients enrolled in the study, one was a relapsed patient (gray dashed line, surgery treatment performed; whose son donated the frozen sample in **Figure 2**), while the other two were treatment-naïve (gray solid lines). **(F)** Boxplots show DNA repair score quantification per sample (above) or per epi-cell.

**Figure 4:**
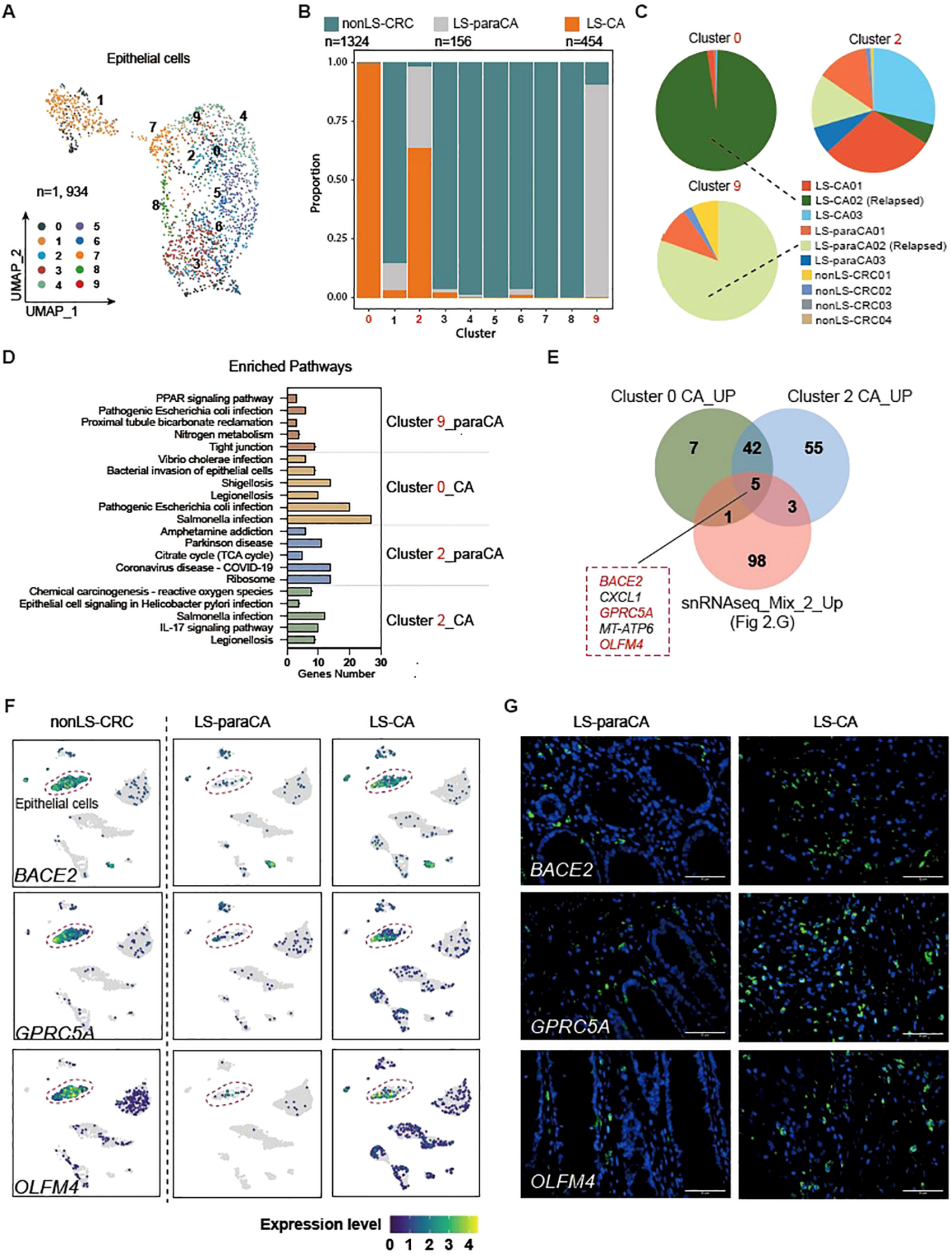
Prioritizing novel epithelial biomarkers for tracing the transition between para-carcinoma and carcinoma in LS (LS-paraCA vs. LS-CA). **(A)** Epithelial cells were filtered out for further analysis and annotated as clusters 0–9 in the UMAP plot. **(B, C)** Bar and pie plots show the contribution of sources to epithelial clusters. Cluster 0 contained cells largely from Lynch CA_02, whereas Cluster 9 was composed of Lynch paraCA_02. The patient who donated Lynch CA_02 and paraCA_02 is identified as a relapsed LS. **(D)** KEGG enrichment analysis showing the enriched bioprocesses in the four epithelial cell clusters. **(E)** Five genes with upregulated expression in LS carcinoma epithelial cells were prioritized based on the scRNA-seq and snRNA-seq datasets. **(F)** Expression of *BACE2, GPRC5A* and *OLFM4* in the paired LS-paraCA and LS-CA groups. Epithelial cells are circled by dashed lines. Note that the expression of genes in epithelial cells from nonLS-CRC samples was also very strong. **(G)** Verification of the low-to-high transition in the expression of *BACE2, GPRC5A* and *OLFM4*. Paraffin sections from paired LS-paraCA and LS-CA samples were prepared, and immune-histo-fluorescence (IHF) staining was performed. The expression of the indicated proteins is labeled in green, whereas the nuclei are stained with DAPI in blue. In total, biopsy samples from 4 patients with LS were used for IHF verification.

**Figure 5:**
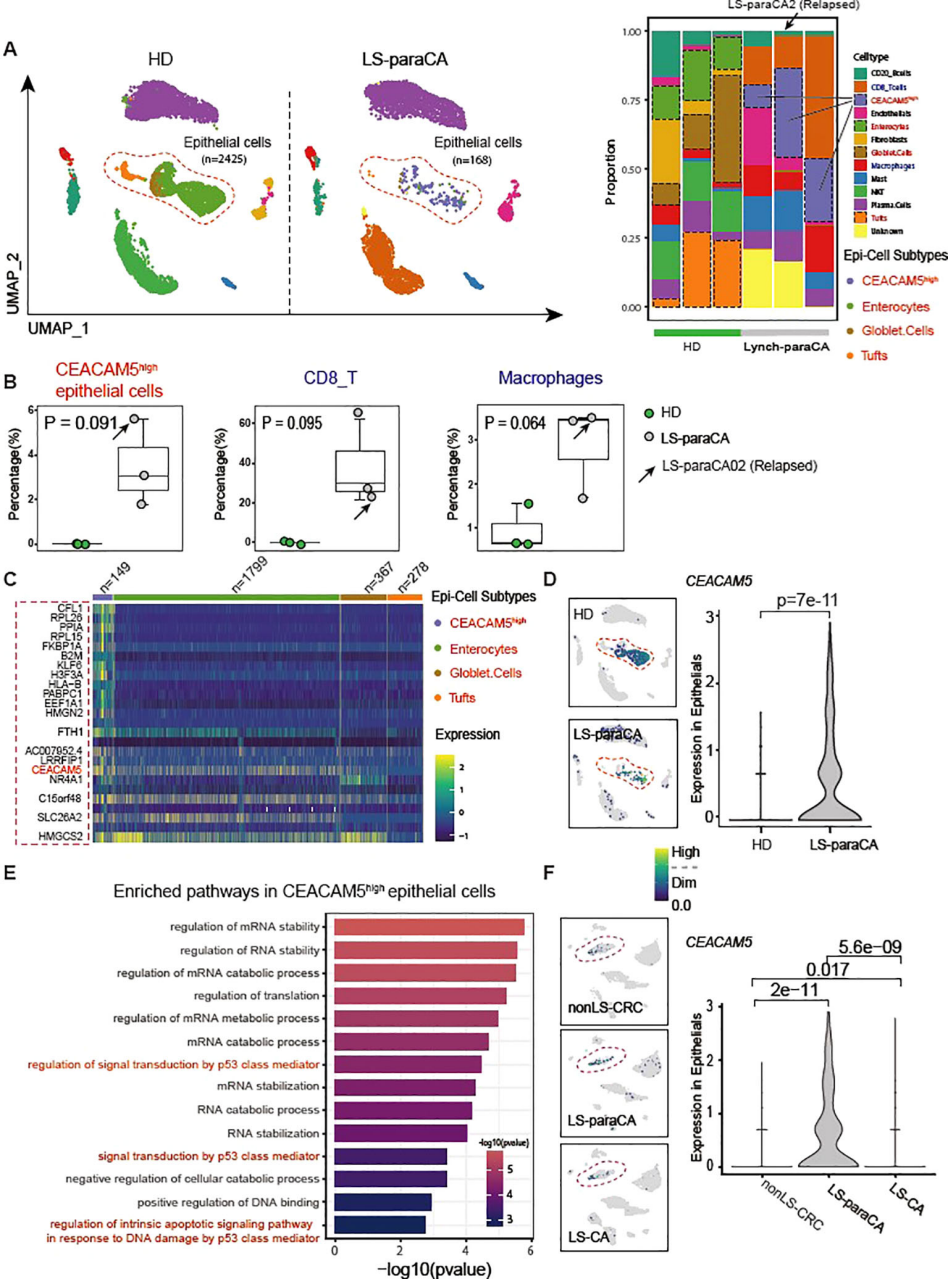
Distinct pre-cancer signs in epithelial and immune cells of LS para-carcinoma. **(A)** The scRNA-seq datasets of LS-paraCA were integrated with those of the colons from healthy donors (HD) (N = 3, respectively; the HD group had 7885 cells in total, whereas the LS-paraCA group had 4766 cells). A dominance of *CEACAM5*^high^ epithelial cells were identified in LS-paraCA. The sample from the patient with relapsed LS is marked with an arrow in the bar plot (left panel). **(B)** The portion of *CEACAM5*^high^ epithelial cells, infiltrated *CD8*_T cells and macrophages was quantified. The sample from the relapsed LS patient is marked with arrows. **(C, E)** Top marker genes co-expressed in *CEACAM5*^high^ epithelial cells and the enriched pathways are illustrated in the gene expression heatmap. Cells from the Enterocytes, Tufts, and Goblet subtypes were used as controls. **(D, F)** Quantification of the expression of *CEACAM5* in epithelial cells of HD and LS-paraCA cells.

**Figure 6:**
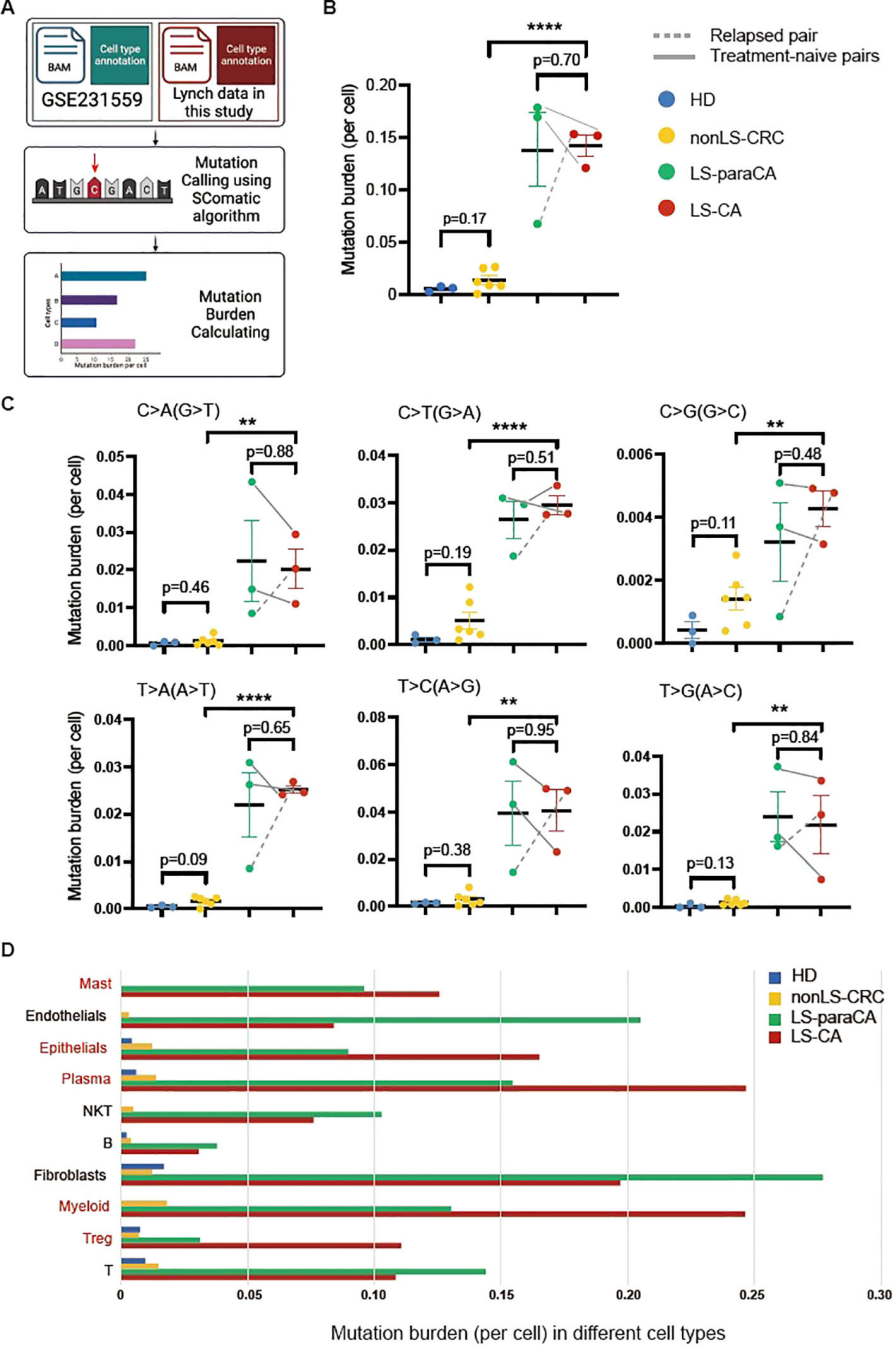
Increased mutation burden in the colon tissues from Lynch syndrome. **(A)** The schematic diagram for calculating mutation burden based on the scRNA-seq datasets using the SComatic algorithm. Only single-base substitutions (SBS) were called in this study. Once mutation calls were achieved, the mutation burden value was quantified by normalizing the total mutation calls to the total cell number in the samples or cell types (See Methods section for details). **(B)** The total SBS mutation burden in the four groups of samples was indicated. **(C)** The six different types of SBS mutation burdens in the four groups of samples as indicated. **(D)** The SBS mutation burdens in different cell types of the four groups were measured and compared. Compared to the LS-paraCA group, the mutation burden appears to be higher in mast, epithelial, plasma, myeloid, and Treg cells in the LS-CA group. **P<0.01, ****P<0.0001.

**Figure 7:**
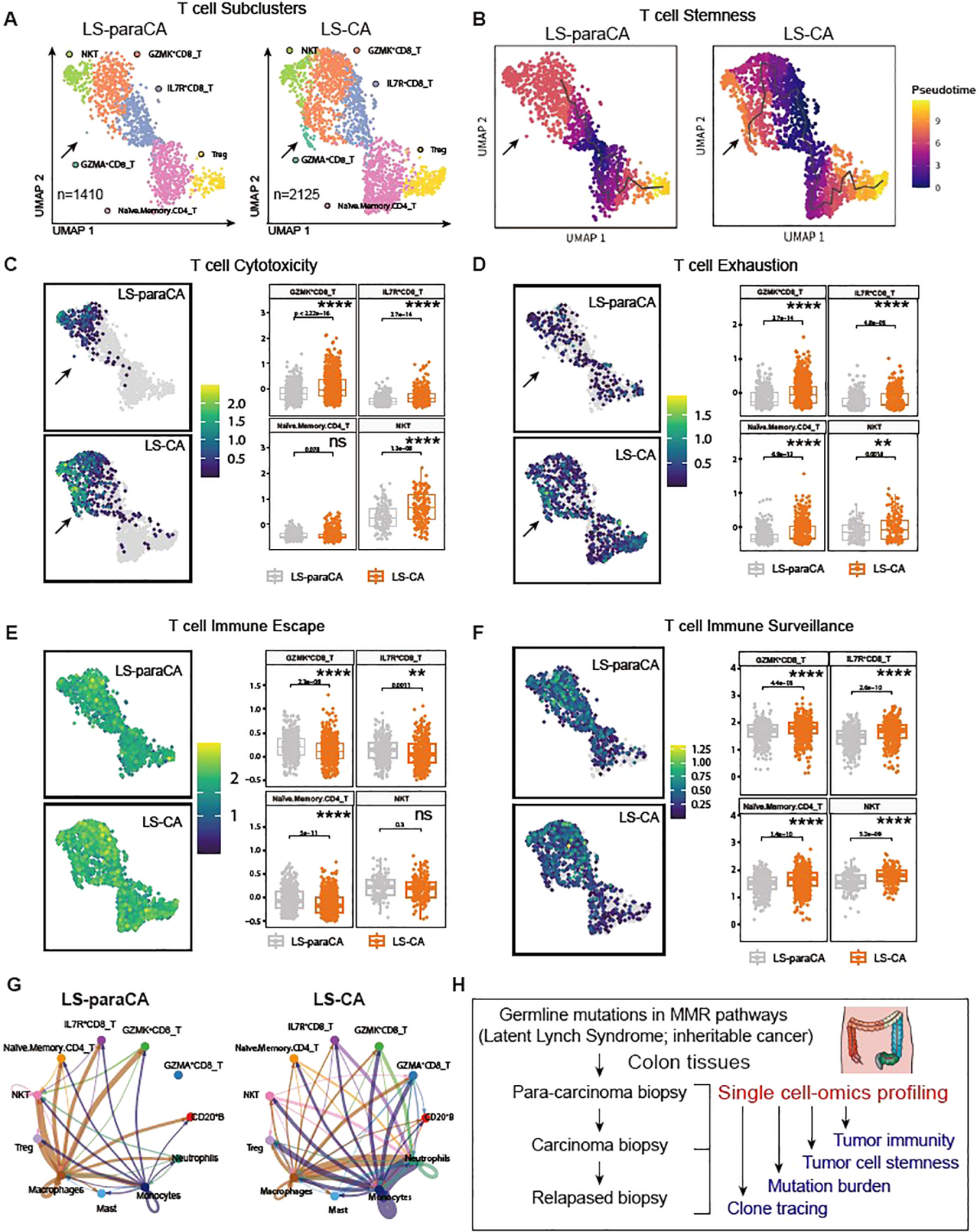
Distinct tumor immunity microenvironment profiles in LS carcinoma. **(A)** T cells from LS-CA and LS-paraCA patients were filtered for further annotation. The LS-paraCA group had n=1410 T cells, whereas the Lynch CA group had n=2125 T cells. Six major T cell subtypes were identified: Naïve *CD4*-T, NKT, Treg, *IL7R*^+^*CD8*_T, *GZMA*^+^*CD8*_T, and *GZMK*^+^*CD8*_T. Note that *GZMA*^+^*CD8*_T cells appear to be more abundant in Lynch CA (arrows). **(B)** The pseudotime trajectory indicating the T cells developed towards two branches: the Naïve *CD4*-T and Treg branches, and *GZMA*^+^*CD8*_T and *GZMK*^+^*CD8*_T branches. *GZMA*^+^*CD8*_T cells appear to be more mature in LS-CA than in LS-paraCA (arrows). **(C-F)** Biological function-instructed scoring of the paired single-cell datasets of T cells from the three LS patients, including T cell cytotoxicity **(C)**, T cell exhaustion **(D)**, T cell immune escape **(E)**, and T cell surveillance **(F)**. **(G)** Circle plots showing interaction profiles across indicated cells in the paired LS samples. The direction of the lines indicates the direction of the cell-cell communication, the width of the lines indicates the interaction strength, and the color of the lines indicates the cell types of the senders. **(H)** Summary of the Study. In this study, we profiled Lynch Syndrome biopsy samples using single-cell or single-nucleus RNA-seq analysis. Paired carcinoma and adjacent para-carcinoma samples were included to facilitate the analysis, including tracing tumor clones, calculating mutation burden, identifying biomarkers for cancer stem cells, and tumor immune microenvironment changes during the transition from pre-carcinoma to carcinoma in the inheritable cancer Lynch Syndrome. See the text in the Discussion section for further detail. **P<0.01, ****P<0.0001.

- Diagnosis of the enrolled LS patients are clearly supported by our pedigree documentations (**Figure 1** and **Supplementary Figure 1**) and briefed in **Table 1**; Colon tissues from HD (n=4), patients with LS (n=6) and with CRC (n=3) were collected for snRNA-seq or scRNA-seq analysis or experimental validations in the study;
- Among the enrolled patients with LS, 1 for snRNA-seq analysis (carcinoma tissue only), 3 for scRNA-seq analysis (paired tissues from para-carcinoma and carcinoma) and 3 for experimental validations; (**Figure 1**)
- Analysis are focused on the comparisons between HD, LS and sporadic CRC; In addition, comparisons between paired carcinoma and para-carcinoma tissues in LS were performed; (**Figures 2**–**7**)
- When HD and LS are compared, a well-known cancer stem cell marker *CEACAM5* is readily detected by snRNA-seq analysis; (**Figure 5**)
- When carcinoma and para-carcinoma from LS patients are compared, both increased immune cell infiltration of and enhanced DNA repair activity in the malignant tissues are readily detected by scRNA-seq analysis; in addition, three upregulated markers in LS carcinoma (*BACE2*, *GPRC5A* and *OLFM4*) were identified and validated experimentally by immune-histo-inflorescence; (**Figures 3**, **4**, **7**)
- The mutation calling analysis with a recently developed package SComatic suggests a comparable mutation burden between carcinoma and para-carcinoma in LS, but such mutation burdens are grossly greater than that from HD; (**Figure 6**)

**Table 1:** Clinical characteristics and brief information of enrolled patients.

Patient index	Sample ID in the study	Biopsy sample used for single cell sequencing	Carcinoma location	TNM stage	Mutations in MMR genes	Immunostaining of MMR protein on resected specimen	Location and ethics`
1	Lynch CA01, Lynch paraCA01	single cell RNA-seq	colorectal	Stage_IIIA	MLH1	MLH1(-), PMS2(-), MSH2(+), MSH6(+)	China, Chinese-Han
2	Lynch CA02, Lynch paraCA02	single cell RNA-seq	colorectal	Stage_IIA	MSH2	MLH1(+), PMS2(+), MSH2(-), MSH6(+)	China, Chinese-Han
3	Lynch CA03, Lynch paraCA03	single cell RNA-seq	colorectal	Stage_IA	MLH1	MLH1(-), PMS2(-), MSH2(+), MSH6(+)	China, Chinese-Han
4	Lyn01*	single nucleus RNA-seq	colorectal	Stage_IB	MSH2	MLH1(+), PMS2(+), MSH2(-), MSH6(+)	China, Chinese-Han
5	NA	NA	colorectal	Stage_IA	MLH1	MLH1(-), PMS2(-), MSH2(+), MSH6(+)	China, Chinese-Han
6	NA	NA	colorectal	Stage_IIB	MSH2	MLH1(+), PMS2(+), MSH2(-), MSH6(-)	China, Chinese-Han

## Introduction

Human babies carrying germline-dominant heterozygous mutations in one of the DNA mismatch repair (MMR) genes, such as *MLH1*, *MSH2*, *MSH6* and *PMS2* (typically loss-of-function mutations), will develop into Lynch Syndrome (LS) in their middle age (~40 years old) and further transform into colorectal cancer (CRC) in the later stages of life (1–4). Because of its close relationship with CRC in the clinic, LS is also known as hereditary nonpolyposis colorectal cancer (HNPCC) according to the Amsterdam criteria, but carriers may also develop extracolonic cancers (1, 5). It has been reported that in the lifetime of carriers, patients with LS are at very high risk of transforming into CRC (40–60%) or endometrial cancer (40–80%) (3, 6).

The drivers or biomarkers for malignant transition from latent LS to full-blown LS or CRC during the ages are largely unknown. It has been proposed that malignant onset could be a result of the accumulation of secondary important mutations (also known as the “second-hit” model). For example, mutations in *APC, TP53* or *KRAS* genes, classic drivers of colon cancers, along with mutations in MMR genes, eventually transform latent LS to malignant CRC (LS-CRC) (3, 4, 6). Moreover, except for differences in gene mutation panels for diagnosis, it is not completely understood if LS-CRC is distinct from or similar to patients with regular and sporadic CRC without LS germline mutations (here labeled as nonLS-CRC) in the pathology of epithelial carcinoma cells and tumor immunity microenvironment (TIME) (1, 3).

In the last decade, CRC biopsy samples have been broadly examined by single-cell omics in multi-clinical centers, including RNA sequencing, ATAC sequencing, and TCR-sequencing (7–11). In some studies, CRC patients with somatic mutations in MMR genes were enrolled as sporadic CRC; however, the small subset of CRC patients, LS-CRC, has never been studied in detail with regard to the evolution trajectory of this rare cancer type and any unique features in tumor immunity at the single-cell level (12).

Nevertheless, single-cell sequencing technologies are powerful assets in addition to traditional pathologies and diagnosis. Single-cell omics studies have revolutionized the research of the transition of malignant cells (13). Even at the most basic level, single-cell sequencing technologies can provide a systematic map (a cell atlas) of tumor progression and alteration of their microenvironment. To specifically profile the cell atlas of LS, a rare heritable cancer, we carried out in parallel single-nuclear RNA-sequencing (snRNA-seq) on a frozen LS colon sample and single-cell RNA-sequencing (scRNA-seq) on three paired fresh LS colon samples (carcinoma versus adjacent para-carcinoma). Through a deep bio-computational analysis at both the mutation and functional levels, in this study, we provided a comprehensive single-cell atlas of LS using paired samples and/or family samples. Dramatic increases in immune cell infiltration and DNA repair activity were observed in LS carcinoma compared to the adjacent normal colon tissue. Using healthy and sporadic CRC samples as negative and positive controls, respectively, we also observed that LS epithelial cells maintained high stemness activity and had a large portion of cancer-stem-cell-like cells marked by Carcino Embryonic Antigen-related Cell Adhesion Molecule 5 (*CEACAM5*). This study highlights important characteristics during the malignant transition in LS.

## Materials and methods

### Ethical approval of human sample acquisition, genetic and pathological diagnosis

This study was conducted with the approval of the Seventh Medical Center of the Chinese PLA General Hospital’s Ethics Committee (Number: 2021-03), and participants provided written informed consent prior to participating and in accordance with the 1964 Helsinki Declaration and its later amendments in the study. Patients with LS were enrolled in the hospital between November 2019 and March 2023 and they are Chinese-Han from Beijing, Henan, Shandong, Shanxi, Inner Mongolia and Hebei provinces, respectively. The diagnosis of LS was based on the Amsterdam II criteria and germline mutations were subsequently verified. Invasive adenocarcinoma staging was determined based on the analyses of surgically resected specimens using the American Joint Committee on Cancer (AJCC) staging system. Pathologists reviewed counterpart sections of all tumor tissues to confirm the diagnosis. Fresh biopsies were obtained by colonoscopy and single-cell suspensions were immediately prepared and subjected to single-cell RNA sequencing analysis. For single nuclear RNA sequencing, tissues were obtained from patients undergoing surgical resection and frozen at -80 degrees. Five of the six patients with LS donated paired samples (LS Carcinoma and adjacent para-Carcinoma). Clinical information and family relationships of the enrolled patients were summarized in **Table 1** (Main text) and **Figure 1A** (Main text; See also completed pedigree information provided in **Supplementary Figure 1**).

### Single-nucleus and single-cell RNA sequencing

Nuclei were isolated from frozen tissues according to the SeekGene standard protocol. After nuclear dissociation, the cDNA library was constructed using the SeekOne library preparation kit. For fresh samples, after digestion, single-cell suspensions were confirmed with the proportion of living cells exceeding 90% and at a proper concentration of cells greater than 1000 cells/μL. cDNA libraries were constructed within 24 hours. We used the double-ended sequencing mode on the Illumina sequencing platform to perform high-throughput sequencing of the constructed libraries.

### Cell clustering, annotation, and visualization

In-house sequencing data analysis was mainly conducted using Linux-Ubuntu Operation System, R language (version: 4.2), Python language (version: 3.8) and has been described in our previous studies (14, 15). Using Seurat (version: 4.1), we conducted standard preprocessing and analysis for snRNA-seq or scRNA-seq datasets. We chose the Uniform Manifold Approximation and Projection (UMAP) to perform dimension reduction and visualize the datasets. Harmony (version 0.1) was used to integrate the datasets and control the batch effect. Clusters were identified using the Seurat cluster-finder computation algorithm. Cell types were further annotated based on the expression of canonical tissue compartment markers.

### Malignant cells identification

In snRNA-seq, we extracted all cells sourced from the LS and obtained the raw count matrix. The R package SCEVAN (version: 1.0) was utilized to classify the malignant cells with count matrix input into the core function ‘pipelineCNA,’ which can also be used to infer the copy number profile of malignant cells and identify sub-clone structures (16).

### Pseudotime analysis

Pseudotime trajectory analysis was accomplished by the R package Monocle (version: 3), which was used to predict the evolution trajectory within each time partition and evaluate differentiation levels across cells, based on the expression patterns of key genes. The trajectory roots were defined at the developmental start points. The cells were colored based on their presumed developmental phases.

### Gene set variation analysis

Gene Set Variation Analysis (GSVA) was used to estimate the variation in pathway activity over a sample population in an unsupervised manner. Through GSVA and irGSVA R packages, we conducted analysis to calculate enrichment score of the 50 classical hallmark signatures from the GSEA molecular signature database (MsigDB).

### Cell-cell communication analysis

Following Seurat analysis of the scRNA-seq datasets, we further analyzed the differential ligand receptors expressed in different groups with the default parameters, which was accomplished using the R package CellChat (Version: 1.5). The algorithm quantized the number of interactions based on the number of ligand-receptor (L-R) pairs across cells, and the interaction weight was also evaluated by removing the effects of the number of cells.

### Construction of gene signatures and module scoring in each cell

We integrated classic gene sets to characterize different bioprocesses and created gene lists for subsequent analyses. The function ‘AddModuleScore’ in the Seurat package was used to score the average expression levels for each cluster. All signatures were binned based on the average expression, whereas the control signatures were randomly selected from each bin.

### Immunofluorescence staining

Paraffin-embedded tissue sections were prepared. Tissue sections were dewaxed with xylene, rehydrated, and subjected to antigen retrieval. The slides were blocked with goat serum and incubated with primary antibodies Rabbit monoclonal against BACE2 (1:50, ab270458, Abcam), rabbit polyclonal to GPRCA5 (1:50, ab155557, Abcam), or rabbit polyclonal to OLFM4 (1:50, ab188822, Abcam) at 4 °C overnight. Secondary goat anti-rabbit IgG conjugated with Cy5 (1:200; Servicebio) was added and incubated at 37°C for 30 min. Nuclei were stained with DAPI. The slides were observed under a laser confocal microscope (Zeiss, Germany).

### Single base substitution mutations calling by SComatic

The biocomputational working platform SComatic is a newly developed tool kit calling somatic mutations in cells, and any germline mutations were filtered out in the procedure (17). Thus, to our knowledge, SComatic is one of the best algorithms for measuring somatic mutations induced by LS MMR mutations. The SBS calling was conducted in the SComatic software according to the tutorial of SComatic (Python scripts in the Linux Miniconda environment). First, raw sequencing files (formatted as BAM, containing aligned sequencing reads for all cell types) were split into cell type-specific BAMs using precomputed cell type annotations in Seurat. Second, the base count information for each cell type and every position in the genome was recorded and merged into a single matrix as TSV files. Finally, variants were called, and high-quality mutations were marked with ‘PASS’ label for downstream statistical analysis. Single base substitutions (SBS) were identified in six types: C>T(G>A), C>G(G>C), C>A(G>T), T>C(A>G), T>A(A>T), and T>G(A>C), and the total SBS number is the sum of these six types of SBS. The mutation burden (per sample or cell type) was normalized by dividing the number of mutations by the number of cells in each sample (or cell type).

### Integration of LS scRNA-seq datasets with the single-cell reference atlas of sporadic CRC

To place our Lynch syndrome scRNA-seq datasets within the broad context of CRC immunity, we integrated our scRNA-seq data with the large-scale CRC atlas published by *Marteau et al.* (BioRxiv-2025, doi:10.1101/2024.08.26.609563). Briefly, we downloaded the processed atlas data (available at https://crc.icbi.at) and mapped our LS single-cell profiles onto the reference latent space using the scANVI algorithm (consistent with the atlas construction). Subsequently, we extracted the subset of samples classified as MSI-high/MMR-deficient CRC. This enabled direct comparison of cell type compositions, identification of shared and unique cell states, and differential expression analysis within matched cell subtypes between LS-CA (N = 3) and the sporadic MSI-high/MMR-deficient cohort (N = 149).

### Statistical analysis

Statistical analyses were performed using Prism 9 and the R software (version 4.2.1). We chose appropriate statistical methods to calculate two-tailed p-values to evaluate significance. For gene expression, |log_2_ fold change | >0.3 and p<0.05, were considered statistically significant. In other comparative analyses, statistical significance was set at p<0.05.

### Data availability

Access to the single-cell RNA sequencing dataset can be made available to anyone through the following link: https://doi.org/10.5281/zenodo.15846754. Due to ethical, privacy or security reasons, the other data in the Main text and **Supplementary Material**s are available upon reasonable request to the Lead contact (us36zcai@tmu.edu.cn).

## Results

### Clinical information of the enrolled LS patients

The clinical information of the LS patients (n=6; 2 male and 4 female) enrolled in the study is briefly described in **Table 1**. A cropped version of the family pedigree is shown in **Figure 1A** (full version in **Supplementary Figure 1**). The six patients with LS were from five families (Chinese-Han from 5 different provinces). The years of hospitalization in our center were between 2019 and 2023, and their ages ranged from 36 to 60 years old. All LS biopsy donors met the Amsterdam II criteria and underwent subsequent verification of germline mutations by sequencing a panel of genes, including the four LS-related MMR genes. Germline mutations in genes other than MMR were ruled out. Single-cell sequencing datasets of healthy controls and CRC without indicated LS diagnosis or LS-related MMR somatic mutations (marked as nonLS-CRC hereafter) were downloaded from the public resource. Of the six LS patients enrolled in the study, two were from a family (**Figure 1A**): the donor of the frozen sample was the son of one of the five paired sample donors, who was diagnosed with a relapsed one (a surgery treatment was performed 8 years ago). One of the six LS patients was diagnosed with endometrial cancer and relapsed LS (surgery treatment was performed 15 years ago), and the other five patients did not report extracolonic cancers. Two different sequencing platforms, snRNA-seq and scRNA-seq, were conducted in the study (**Figure 1B**). As briefed in **Figure 1C**, comparisons were designed to characterize the transition between non-malignancies and malignancies in LS (LS-paraCA vs. LS-CA) or between healthy state and pre-malignancies (Healthy vs. LS-paraCA).

### Analysis of a frozen LS colon tissue by snRNA-seq

The frozen malignant colonic tissue from a LS patient (Sample ID in the study: Lynch_01) was used for single nucleus RNA sequencing (snRNA-seq) analysis. Datasets of snRNA-seq of four healthy donors were integrated with Lynch_01 as a control (Sample ID in the study: HB01301, HB01401, HB01406, and HB01501) (10). After standard quality control, 32,083 nuclear transcriptomes were used for downstream analyses. Three major cell types, epithelial cells, stromal cells, and immune cells, were annotated. The Lynch_01 sample exhibited an increase in epithelial cells compared to controls but had much fewer stromal and immune cells in the frozen sample (in Lynch_01, the percentage of epithelial cells was 95.3%, while that of stromal and immune cells was 3.6% and 1.1%, respectively).

To characterize the alterations in the frozen LS samples, only epithelial cells were used for further downstream analysis (**Figure 2A**; the total epithelial cell number was 11353 and Lynch_01 had 3980 cells). As shown in **Figures 2A–D**, clusters 0 to 9 were annotated by the standard Seurat analysis, while tumor and non-tumor cells were inferred from the epithelial cell pool based on copy number variants (CNVs) using the SCEVAN algorithm (16). Epithelial clusters 2, 3, 5, 8, and 9 had relatively low proportions of tumor-like cells. However, clusters 0, 1, 4, 6, and 7 exhibited a high proportion of tumor-like cells, and these clusters were renamed Mix_1 to 5, as indicated (**Figure 2B**). Of note, most of the cells in Lynch_01 were recognized as tumor-like cells (tumor cells: 83.3%; non-tumor cells: 16.7%), and three clones and their phylogenetic tree are indicated (**Figure 2C**). Although the phylogenetic tree suggested that Clone_1 and Clone_2 were more correlated, the ratios of the three clones in the epithelium and their lineages in the epithelial cell subtypes were comparable, suggesting that no dominant clones were observed during the genesis of the eight subtypes of epithelial cells (i.e. Mix 1 to 5, *BEST4*^high^ enterocytes, enterocytes, and goblet cells; **Figure 2D**).

Interestingly, the expression of numerous markers and pseudotime analysis suggested that the pool of Mix_2 epithelial cells (Cluster 7) was dominated by intestinal stem cells (**Figures 2E, F**). Five gene signatures, including the well-known *LGR5*, are also recognized as intestinal cancer stem cell marker genes (19–23). Quantification of Mix_2 in the samples suggested that Lynch_01 had a much higher proportion of Mix_2 epithelial stem cells than the four healthy controls (16.2% in Lynch_01 vs. 4.0% in HC). Furthermore, almost all Lynch_01 cells in Mix_2 were inferred as malignant cells (tumor-like cells; 643 cells out of 645 cells). The proportion of Clone_3 in Mix_2 was the highest (Clone_3,65.2%; Clone_2,27.2%; Clone_1,7.3%; non-malignant, 0.3%) (**Figure 2D**). Differentially expressed gene (DEG) analysis in the Mix_2 pool revealed that genes, including *DST* and *MAGI*, were upregulated in the tumor-like sub-pool, while genes, including *LINGO1* (*Leucine-rich repeat and Ig domain protein 1*) and *ACTB*, were downregulated in the tumor-like subgroup compared to those in the non-tumor-like sub-pool (**Figure 2G**). Interestingly, *LINGO1* is reported as a unique and specific biomarker and drug target for the treatment of Ewing sarcoma, suggesting it potentially acts a new marker for LS too. Accordingly, we also revealed that Mix_2, 4, and 5 pools (dominated by the tumor-like cells from Lynch_01) exhibit much higher activity in DNA repair and MYC_ target pathways (**Figure 2H**), strongly suggesting the MMR pathway and malignant activity in Lynch_01 compared to healthy controls.

### A cell atlas of LS carcinoma and para-carcinoma revealed by single cell sequencing

To overcome the limitations posed by the absence of immune cells in snRNA-seq analysis, we conducted single-cell RNA sequencing analysis (scRNA-seq) using paired fresh carcinoma samples and their adjacent tissues from three LS patients. Datasets of three sporadic nonLS-CRC patients were included as malignant controls (the 3 datasets were randomly selected from GSE166555) (9). As shown in **Figure 3A**, both LS carcinoma and para-carcinoma samples had major cell types, including stromal cells, epithelial cells, and infiltrated immune cells (B cells, myeloid cells, and T cells). The expression of the classic markers is shown in **Figure 3B**. The cells were further annotated into 16 cell types, as shown in **Figure 3C**. The portion between sample groups is shown in **Figure 3D**. Intestinally, increased infiltration of many types of immune cells was observed in LS carcinoma (LS-CA) compared with para-carcinoma (LS-paraCA) (LS-CA vs. LS-paraCA) (**Figure 3E**). Of note, among the three LS patients, the relapsed donor always exhibited a pattern of increased infiltration of neutrophils, monocytes, macrophages, *GZMA*^+^*CD8*_T, and *GZMK*^+^*CD8*_T, suggesting that LS carcinoma has an increased immune response compared to latent LS-paraCA tissue (dashed lines, **Figure 3E**). Results in **Figure 3E** suggest that immune cells can sense the abnormalities and neoantigens in LS-CA tissues which induce infiltration of immune cells of not only myeloid-lineage but also lymphoid lineage.

From the public resource [Marteau et al. (doi:10.1101/2024.08.26.609563)], we collected scRNA-seq datasets of sporadic CRC patients which are not diagnosed as LS but may carried LS-related somatic mutations in the genes related to the MMR pathway. Clinically, this kind of CRC is also known as Microsatellite Instability [MSI]-High/MMR-deficient CRC. In supporting the indications of **Figure 3E**, we performed an alternative analysis to minimize the batch effect between CRC and LS samples (**Supplementary Figures 2A–C**). Results in **Supplementary Figure 2C** suggest that a high infiltration rate of granulocytes/neutrophils in LS-CA, compared to MMR-deficient nonLS-CRC. We therefore integrated the granulocytes/neutrophils of the two groups for further analysis on the DEGs and enriched pathways (**Supplementary Figures 2D–F**). In summary, LS-CA appears to have higher grade of granulocyte infiltration compared to MSI-high/MMR-deficient CRC. Accordingly, when DNA repair activity was measured in the cell atlas, we observed that the activity was increased in LS-CA compared to that in LS-paraCA (**Figure 3F**). These results demonstrate that the study provides for the first time a cell atlas for comparing LS carcinoma and para-carcinoma, and in the atlas, infiltration of immune cells and DNA repair activity are readily detected at a higher level in LS carcinoma than that in LS para-carcinoma.

### Prioritizing novel biomarkers tracing the malignant transition in the paired LS epithelial cells

Although there were not enough epithelial cells available to perform similar clonal analysis by SCVAN in each LS paired sample, we were able to filter out epithelial cells and compare the expression of important genes between LS-CA and LS-paraCA. Seurat analysis of the epithelial cells revealed that 10 different clusters were recognized and that Clusters 0, 2, and 9 were dominated by LS samples (**Figures 4A–C**). We enriched the biological pathways in these clusters using KEGG analysis (**Figure 4D**). Furthermore, we prioritized five conserved genes that were upregulated in Mix_2 of snRNA-seq (**Figure 2G**) and in Cluster_0_CA and Cluster_2_CA: *BACE2*, *CXCL1*, *GPRC5A*, *MT-ATP6* and *OLFM4* (**Figure 4E**). As *CXCL1* encodes a secreted protein and *MT-ATP6* encodes a mitochondrial protein, we chose the other three genes to verify whether their expression is dominant in epithelial cells and to perform protein-level verification using immune-histo-fluorescence (IHF) experiments. As shown in **Figures 4F, G**, the expression of *BACE2*, *GPRC5A* and *OLFM4* and their encoded proteins were much higher in malignant CRC and LS-CA tissues, while their expression in LS-paraCA tissues was limited (quantification of the IHF staining is shown in **Supplementary Figure 3**). In summary, these results demonstrate that LS patients have a unique epithelial cell cluster distinct from CRC tissue and that a series of conserved biomarkers should be used to trace malignant transition in LS.

### *CEACAM5* as an early biomarker for abnormal epithelial cells in latent LS samples

LS is a disease induced by germline mutations, and the malignant sign is probably much earlier than the appearance of carcinoma tissue. This suggests that the epithelial cells from LS-paraCA may show some abnormalities when compared to healthy controls. To test this hypothesis, we then integrated our datasets of LS-paraCA epithelial cells with those of three healthy donors (GSE166555) to determine whether markers earlier than the above three biomarkers exist (9). As shown in **Figures 5A–C**, a *CEACAM5*^high^ epithelial cell cluster was recognized based on the single-cell atlas, and it appears that such a cluster is specific to LS patients. *CEACAM5* is well-known cancer stem cell biomarker and immune checkpoint target (24, 25). Interestingly, the proportion of *CD8*^+^ T cells and macrophages, two important immune cells in the tumor microenvironment, was much higher in the LS-paraCA group than in the healthy controls (HD) (**Figure 5C**). The genes that were highly co-expressed with *CEACAM5* were plotted in a gene expression heatmap, and the enriched pathways included RNA stability, metabolic-related processes, and *p53*-related DNA damage pathways (i.e. signal transduction by p53 class mediators and apoptosis pathway related to p53 DNA damage and repair) (font in red, **Figures 5C, E**). Given that LS mutations are DNA damage and repair pathway-related, the observation of the *p53*-related DNA damage pathways suggests a high quality of our scRNA-seq dataset. As *CEACAM5* expression is not specific to *CEACAM5*^high^ epithelial cells and LS patients, we quantified the level of *CEACAM5* in healthy controls, LS-paraCA, LS-CA, and nonLS-CRC patients as controls. As shown in **Figure 5D**, healthy controls only maintained a very dim expression level of *CEACAM5* in epithelial cells, while LS-paraCA manifested a much higher expression of that. Similarly, *CEACAM5* was expressed in nonLS-CRC and LS-CA cells (**Figure 5F**). Taken together, these results suggest that LS-paraCA may confer biomarkers such as *CEACAM5* and infiltration of immune cells much earlier than previously thought. These biomarkers should be tracked along with LS progression as early as possible to monitor malignant transformation in patients’ management.

### Comparable high SBS mutation burden in LS carcinoma and para-carcinoma at the coding-sequence-wide level

The “second-hit” model has been proposed to explain the malignant transition in the LS, indicating LS carcinoma tissue may have an increased tumor mutation burden than that in the LS para-carcinoma tissues. To test if this hypothesis is true, we measured the single-base substitution (SBS) mutation burden at the coding-sequence wide (rather than genome-wide) using the SComatic algorithm tool. The computational analysis procedure is illustrated in **Figure 6A** (see reference for details) (17). To test whether any differences in SBS burden exist between LS and non-LS CRC, datasets of three healthy controls and six CRC patients were also included (GSE231559) (18). As shown in **Figure 6A**, the SBS mutation burden was greater than 10 folds higher in LS-CA and LS-paraCA than in that in nonLS-CRC (average fold change: 10.7; p<0.001). As expected, the mutation burden in nonLS-CRC patients was also much higher than that in healthy controls but the difference did not appear to be significant (fold change on average: 1.7; p=0.17). LS-CA and LS-paraCA maintained a high SBS mutation burden; however, no significant differences were detected between LS-CA and LS-paraCA at the total level or the level of each single SBS pattern (fold change in average: 1.1, p=0.7, **Figure 6B**). We also measured the differences of SBS mutation burden in the six main types, and still no significant differences were observed (**Figure 6C**). Notably, the relapsed LS patient (dashed line, **Figures 6B, C**) always exhibited an increased SBS mutation burden in the CA tissue compared to that in the paraCA tissue. Furthermore, mast, epithelial, plasma, myeloid, and Treg cells appeared to have a higher mutation burden in LS-CA than that in LS-paraCA (**Figure 6D**). In summary, these results demonstrate that LS maintains a comparably high level of SBS mutation burden, but no significant difference was detected between carcinoma and para-carcinoma tissues.

### Tumor immunity is mobilized during the transition from latent LS to full-blown LS-CRC

The cell atlas of LS also facilitates detailed profiling of alterations in the TIME of heritable cancer. As shown in **Figures 3E, B**, we demonstrated that immune cells are dramatically mobilized when comparing LS-CA and LS-paraCA or comparing LS- paraCA and healthy controls (HD). To dissect the molecular features of tumor immunity in detail in patients with LS, we isolated T cells for further analyses (**Figure 7A**). Pseudotime trajectory analysis revealed T cells branching into *CD4* and *CD8* categories, with Naive Memory *CD4*^+^T cells evolving into Treg cells, and *IL7R*^high^*CD8*^+^T cells transitioning into *GZMK*^high^*CD8*^+^T, *GZMA*^high^*CD8*^+^T, and NKT cells (**Figure 7B**). Functional assessment of T cells using established canonical gene sets indicated consistent trends in LS carcinoma (LS-CA vs. LS-paraCA), with enhanced cytotoxicity, exhaustion, and immune surveillance across T cell subtypes (**Figures 7C–F**). Furthermore, the overall profiles of cell-cell interaction strength were more complex and abundant in LS-CA than in that in LS-paraCA (**Figure 7G**). In summary, these results demonstrate that immune cells were more actively mobilized into the malignant tissue in LS and that immune cell-cell communication was also more active in malignant LS tissue (26).

## Discussion

In this study, we focused on a specific group of colorectal cancer patients diagnosed with Lynch Syndrome and profiled the malignant transition using single-cell RNA sequencing and single-nuclear RNA sequencing.

As outlined in **Figure 7H**, our study demonstrated that: 1) both frozen colon samples and fresh samples are feasible for single-cell (or nucleus) analysis (median UMI numbers are between 1120 and 2638 with snRNA-seq having the lowest UMI); 2) DNA repair activity is readily detected at the gene expression level when epithelial cells of LS are compared with normal epithelial cells; 3) SBS mutation burden is comparable in LS-CA and LS-paraCA but greater than that in CRC or healthy donors; 4) LS carcinoma has a high stemness score and numerous biomarkers including *CEACAM5* (already known), *BACE2*, *GPRC5A* and *OLFM4* (newly identified here), which may be utilized as novel molecular markers for tracing the malignant transition for monitoring LS in future; 5) The infiltration and activity of immune cells are greatly mobilized during the transition from a non-malignant state to a malignant state in the LS. In conclusion, this study sets up a comprehensive computational workflow for observing neoplasm transition in the real world for LS by utilizing a single-cell sequencing dataset.

### Limitations of the study

#### Limitations of acquired clinical samples

An obvious limitation of the present study is that only four LS patients were enrolled for a single-cell omics study plus two additional LS donors for experimental verification, and no longitudinal biopsy samples were included. Considering that clinical studies rely on biopsy sample availability in practice, the six donors represent a small LS population. Nonetheless, this study is certainly instructive to the field since we are using single-cell sequencing technologies to monitor the transition (or the difference) between latent LS colon tissue and diseased (malignant) LS colon tissue. Importantly, two members of a family with LS were enrolled in the study (a mother and a son), and the mother was a relapsed LS patient, while the other three LS patients were treatment-naive. This diversified representation of the enrolled LS cohort with the scRNA-seq dataset represents an invaluable resource for future LS studies.

Another obvious limitation of the present study is that no whole-exome-sequencing (WES) analysis was conducted to validate the findings, as we only used the availability of the scRNA-seq dataset to compare the mutation burden of LS-CA and LS-paraCA (27). Reutilization of the scRNA-seq dataset by the SComatic algorithm enabled us to compute the SBS mutation burden without additional sequencing costs. The addition of WES sequencing to paired samples of colon tissue or single-cell DNA sequencing offers an opportunity to observe the mutation burden at the whole-genome and single-cell levels.

#### Limitations of our bio-computation analysis and the future direction of the study

The present study largely relies on the computational analysis of scRNA-seq or snRNA-seq datasets with transcriptomic features and a large number of cells. Annotation of the single cells with a regular analysis protocol on the Seurat platform enabled us to identify the changes in the proportion of cell types and cell-cell communications. Using SCEVAN and SComatic algorithms, we were also able to profile CNV alterations and SBS mutations. These two algorithms are particularly important because LS is a heritable CRC with mutations in MMR genes. Furthermore, we validated our findings using immunohistochemistry and flow cytometry experiments. In addition, considering that the current scRNA-seq dataset is only about 150 bp of 3’ regions of expressed genes, full-length sequencing of the cDNA will be more informative for the SComatic analysis. If the mutation burden of LS-paraCA and LS-CA is indeed comparable, epi-mutation analysis should be considered in the future. By the way, combination of scRNA-seq and scATAC-seq will assist the cell atlas study of LS as well. We envision that an improved bio-computational analysis working-flow will be instructive for future LS studies when more donors are enrolled.

## Conclusion

Taken together, this study provides a cell atlas of colon diseased tissues from LS patients at a single-cell level. Through paired samples, we profiled the alterations in cancer stem cell markers and infiltrated tumor immune cells during the transition of latent LS and full-blown LS. Based on a limited number of enrolled patients, we did not observe a dramatic difference in SBS during the transition; however, an increase in DNA repair activity was detected. This study suggests several novel cancer stem cell biomarkers for monitoring LS progression and suggests that single-cell sequencing technologies empower a deeper understanding of the pathologies in LS, the heritable colorectal cancer.

## Data Availability

The datasets presented in this study can be found in online repositories. The names of the repository/repositories and accession number(s) can be found below: https://ngdc.cncb.ac.cn/gsa-human/browse/HRA006527, PRJCA022913.
