# Supplementary material for: Tracing the stemness and malignant transition in a heritable colorectal cancer Lynch Syndrome by single-cell RNA-seq analysis
Source: Front Immunol. 2026 May 25;17:1722806. doi: 10.3389/fimmu.2026.1722806 (PMC13243404; doi:10.3389/fimmu.2026.1722806)
Supplement: Supplementary file 1 [file DataSheet1.pdf]

Supp. Fig. 1

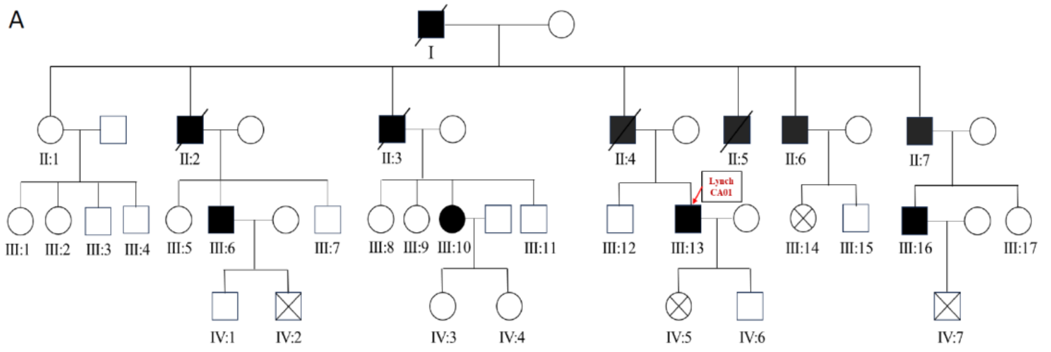

Notes:
I: diagnosed with CRC at 32 y and died at 34 y.
II:2: with MLH1 germline mutation, diagnosed with pancreatic cancer at 36 y and died at 37 y.
II:3: with MLH1 germline mutation, diagnosed with CRC (MSI) at 35 y and died at 39 y.
II:4: with MLH1 germline mutation, diagnosed with CRC (MSI) at 32 y and died at 36 y.
II:5: with MLH1 germline mutation, diagnosed with pelvic ureteral cancer at 28 y and died at 28 y.
II:6: with MLH1 germline mutation, diagnosed with CRC (MSI) at 43 y and had surgery.
II:7: with MLH1 germline mutation, diagnosed with CRC (MSI) at 51 y and had surgery.
III:6: with MLH1 germline mutation, diagnosed with CRC (MSI) at 45 y and had surgery.
III:10: with MLH1 germline mutation, diagnosed with endometrial cancer at 42 y and had surgery.
III:13: with MLH1 germline mutation, diagnosed with CRC (MSI) at 47 y and had surgery.
III:14: with MLH1 germline mutation, but is currently disease-free (46 y).
III:16: with MLH1 germline mutation, diagnosed with CRC (MSI) at 39 y and had surgery.
IV:2、IV:5 and IV:7: with MLH1 germline mutation, but are currently disease-free (38 y、22 y and 24 y).

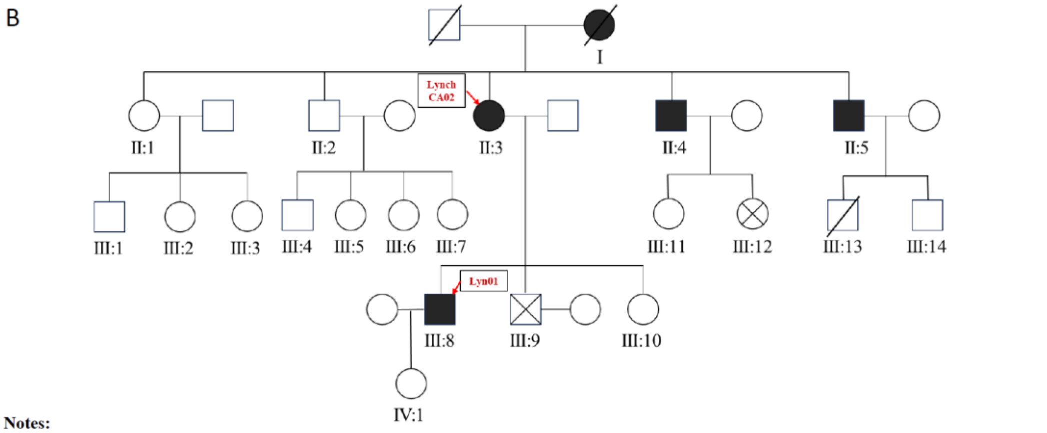

Notes:
I: diagnosed with CRC (MSI) at 38 y and died at 40 y.
II:3: with MSH2 germline mutation, diagnosed with CRC (MSI) at 48 y and had surgery, **but recurred** at 59 **y and had** surgery again.
II:4: with MSH2 germline mutation, diagnosed with CRC (MSI) at 50 y and had surgery; diagnosed with Advanced adenoma at 58 y and had ESD under colonoscopy.
II:5: with MSH2 germline mutation, diagnosed with Advanced adenoma at 55 y and had ESD under colonoscopy.
III:8: with MSH2 germline mutation, diagnosed with CRC (MSI) at 38 y and had surgery.
III:9: with MSH2 germline mutation, but is currently disease-free (39 y).
III:12: with MSH2 germline mutation, but is currently disease-free (32y).
III:13: died in a traffic accident at 32 y.

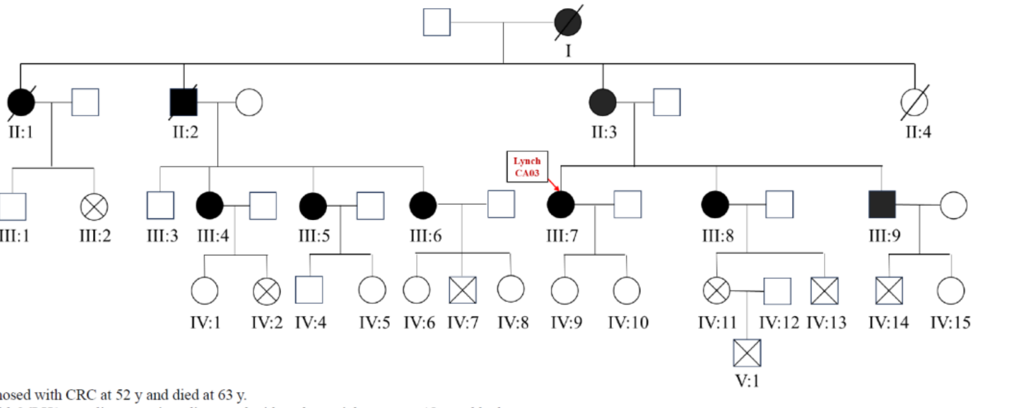

Notes:
I: diagnosed with CRC at 52 y and died at 63 y.
II:1: with MLH1 germline mutation, diagnosed with endometrial cancer at 45 y and had surgery, but died at 67 y.
II:2: with MLH1 germline mutation, diagnosed with CRC (MSI) at 46 y and had surgery, but died at 55 y.
II:3: with MLH1 germline mutation, diagnosed with CRC (MSI) at 58 y and had surgery.
II:4: diagnosed with leukemia at 18 y and died at 19 y.
III:2: with MLH1 germline mutation, but is currently disease-free (53 y).
III:4: with MLH1 germline mutation, diagnosed with endometrial cancer at 39 y and had surgery.
III:5: with MLH1 germline mutation, diagnosed with endometrial cancer at 43 y and had surgery.
III:6: with MLH1 germline mutation, diagnosed with endometrial cancer at 34 y and had surgery.
III:7: with MLH1 germline mutation, diagnosed with CRC (MSI) at 60 y and had surgery.
III:8: with MLH1 germline mutation, diagnosed with ovarian cancer at 47 y and had surgery.
III:9: with MLH1 germline mutation, diagnosed with CRC (MSI) at 42 y and had surgery.
IV:2、IV:7、IV:11、IV:13 and IV:14: with MLH1 germline mutation, but are currently disease-free (35 y、31 y、27 y、19 y and 20 y).
V:1: with MLH1 germline mutation, but is currently disease-free (6 y).

Supp. Fig. 1 Uncropped version of the three family pedigrees of patients with Lynch syndrome. Related to **Figure 1A.**

(**A**) Family pedigree of Lynch CA01.

(**B**) Family pedigree of Lynch CA02 and Lyn01.

© Family pedigree of Lynch CA03.

All cases included in the study met the Amsterdam II criteria: 1) three relatives with Lynch Syndrome-related cancers; 2) one of which is a first-degree relative of the other two; 3) Lynch Syndrome-related cancer affects more than one generation; and 4) at least one Lynch Syndrome-related cancer diagnosed before the age of 50 years. Lynch Syndrome-related cancer sites included the colon, rectum, endometrium, ovary (including fallopian), sebaceous carcinoma, small bowel, ureteric, or CNS gliomas (including glioblastoma and astrocytoma).

Supp. Fig. 2

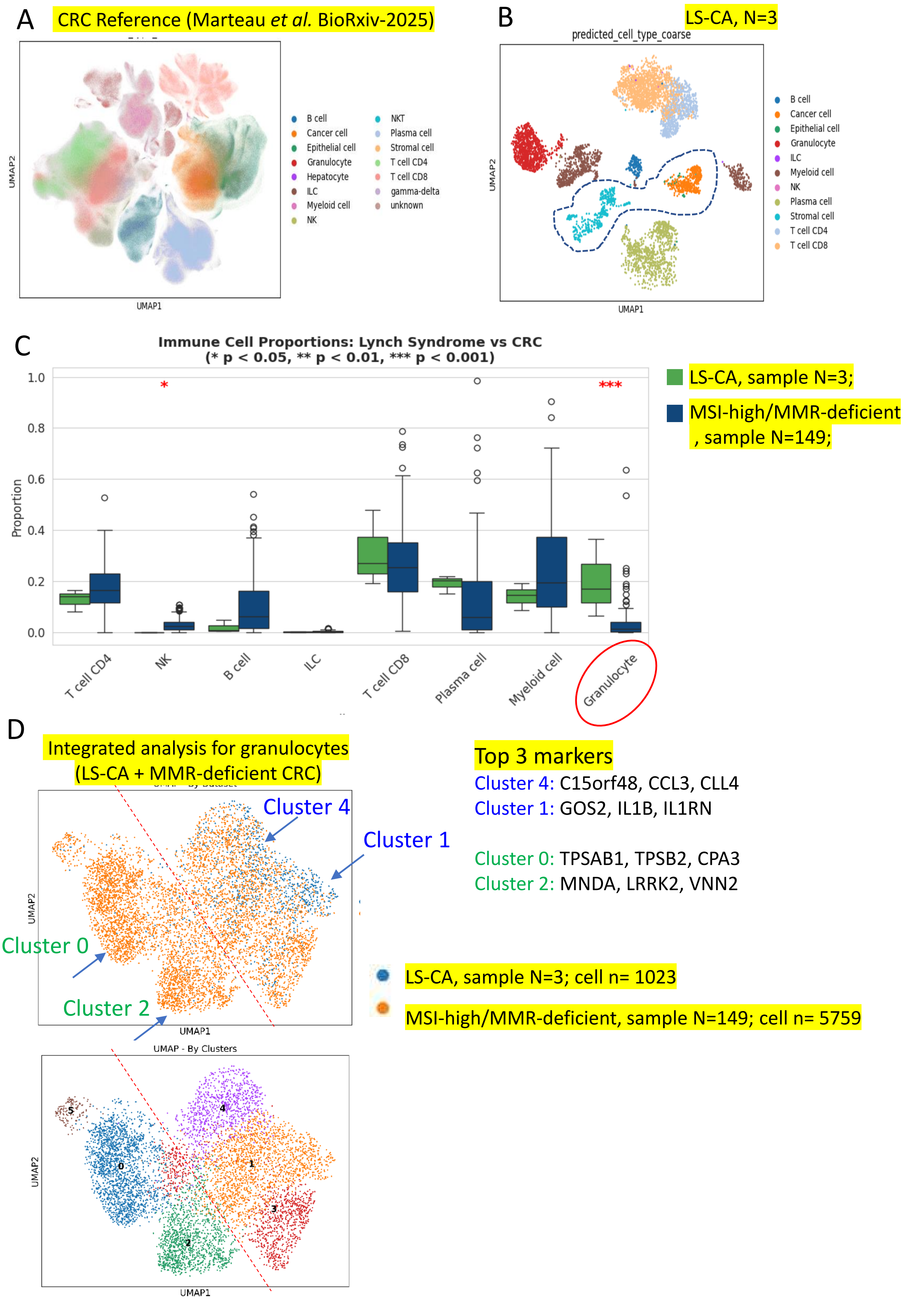
A
CRC Reference (Marteau et al. BioRxiv-2025)
UMAP2
UMAP1
B cell
Cancer cell
Epithelial cell
Granulocyte
Hepatocyte
ILC
Myeloid cell
NK
NKT
Plasma cell
Stromal cell
T cell CD4
T cell CD8
gamma-delta
unknown
B
LS-CA, N=3
predicted_cell_type_coarse
UMAP2
UMAP1
B cell
Cancer cell
Epithelial cell
Granulocyte
ILC
Myeloid cell
NK
Plasma cell
Stromal cell
T cell CD4
T cell CD8
C
Immune Cell Proportions: Lynch Syndrome vs CRC
(* p < 0.05, ** p < 0.01, *** p < 0.001)
Proportion
1.0
0.8
0.6
0.4
0.2
0.0
*
***
T cell CD4
NK
B cell
ILC
T cell CD8
Plasma cell
Myeloid cell
Granulocyte
LS-CA, sample N=3;
MSI-high/MMR-deficient , sample N=149;
D
Integrated analysis for granulocytes (LS-CA + MMR-deficient CRC)
Cluster 4
Cluster 1
Cluster 0
Cluster 2
UMAP2
UMAP1
Top 3 markers
Cluster 4: C15orf48, CCL3, CLL4
Cluster 1: GOS2, IL1B, IL1RN
Cluster 0: TPSAB1, TPSB2, CPA3
Cluster 2: MNDA, LRRK2, VNN2
LS-CA, sample N=3; cell n= 1023
MSI-high/MMR-deficient, sample N=149; cell n= 5759
UMAP - By Clusters
0
1
2
3
4
5
UMAP2
UMAP1

E

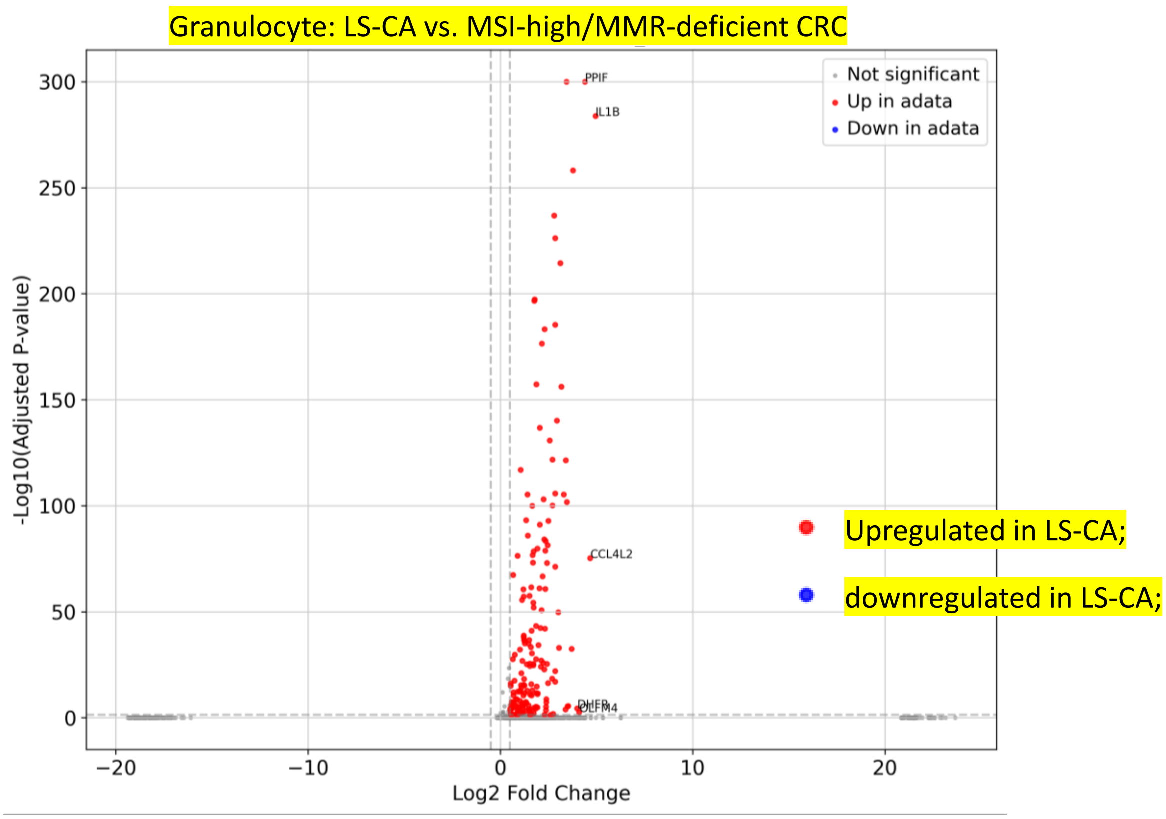
Granulocyte: LS-CA vs. MSI-high/MMR-deficient CRC
Not significant
Up in adata
Down in adata
PPIF
IL1B
CCL4L2
-Log10(Adjusted P-value)
Log2 Fold Change
300
250
200
150
100
50
0
−20
−10
0
10
20
Upregulated in LS-CA;
downregulated in LS-CA;

F

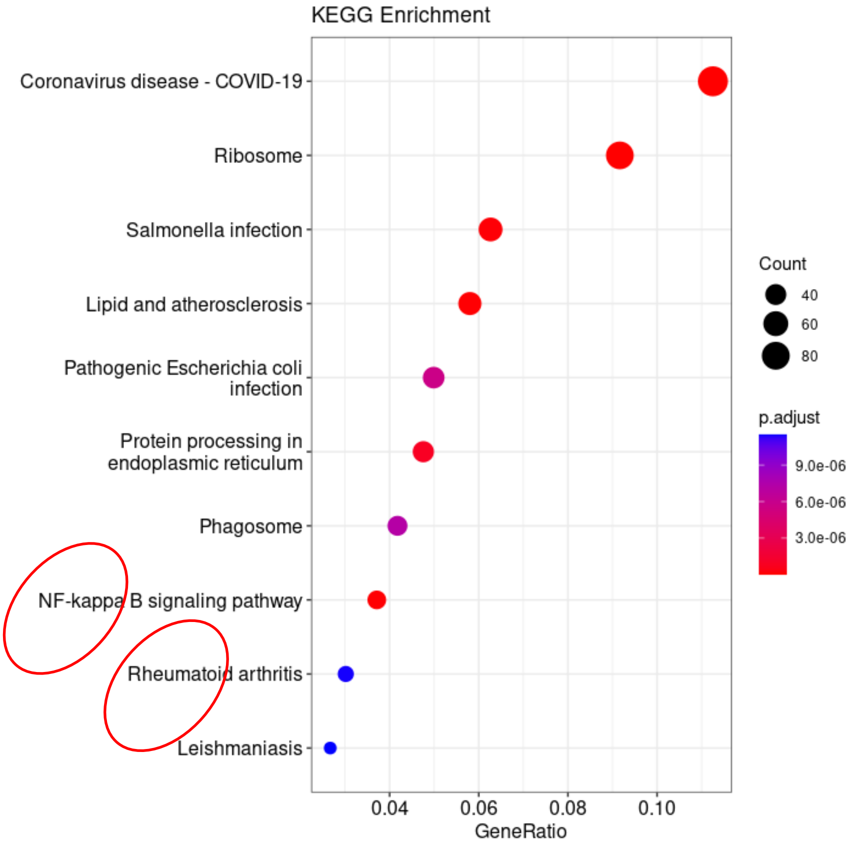
KEGG Enrichment
Coronavirus disease - COVID-19
Ribosome
Salmonella infection
Lipid and atherosclerosis
Pathogenic Escherichia coli infection
Protein processing in endoplasmic reticulum
Phagosome
NF-kappa B signaling pathway
Rheumatoid arthritis
Leishmaniasis
0.04
0.06
0.08
0.10
GeneRatio
Count
40
60
80
p.adjust
9.0e-06
6.0e-06
3.0e-06

Supp. Fig. 2 Comparison of tumor immunity between LS-CA and sporadic MSI-high/MMR-deficient CRC. Related to **Figure 3E.**

(A) Reference CRC dataset and its UMAP were used for annotating the cell types of the LS-CA datasets (N=3) and MSI-high/MMR-deficient CRC (N=149).
(B) Cancer & stromal cells (circled) and immunological cells are annotated in a new UMAP for the LS-CA datasets, suggesting a high quality of our LS-CA/LS-paraCA datasets in the study.
(C) Comparison of the immunological cell compartments between LS-CA and MSI-high/MMR-deficient CRC.
(D) Neutrophils of two groups were extracted for further analysis. As shown in the UMAP, neutrophils of the LS-CA group are dominantly distributed in Cluster 4 and Cluster 1.
(E) Enhanced expression of *PPIF* and *IL1B* is illustrated in the volcano plot.
(F) Enriched bioactivities in the neutrophils of the LS-CA group includes the NFκB inflammatory and infection response pathways.

Supp. Fig. 3

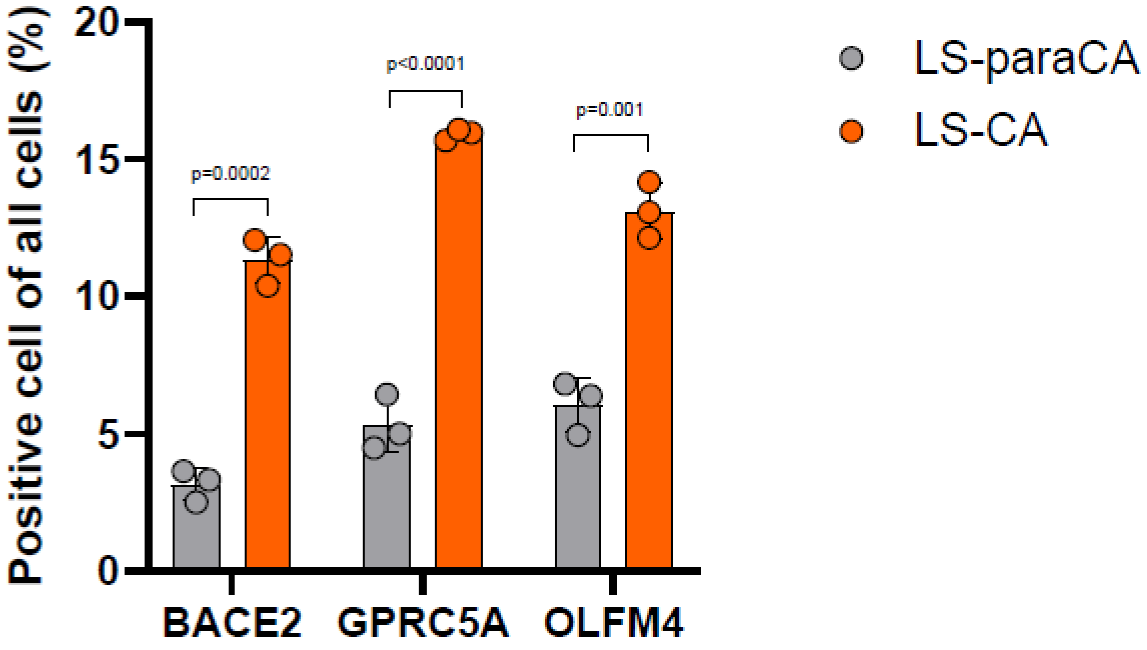

Supp. Fig. 3 Quantification of BACE2, GPRC5A and OLFM4 staining. Related to **Figure 4G**.
